# Novel region discovery method for Infinium 450K DNA methylation data reveals changes associated with aging in muscle and neuronal pathways

**DOI:** 10.1111/acel.12159

**Published:** 2013-10-22

**Authors:** Mei-Lyn Ong, Joanna Dawn Holbrook

**Affiliations:** Singapore Institute for Clinical Sciences, Agency for Science Technology and Research (A*STAR), Brenner Centre for Molecular Medicine30 Medical Drive, Singapore, 117609, Singapore

**Keywords:** aging, epigenetics, EWAS, Infinium, methylation, sarcopenia

## Abstract

We describe a methodology for detecting differentially methylated regions (DMRs) and variably methylated regions (VMRs), in data from Infinium 450K arrays that are very widely used in epigenetic studies. Region detection is more specific than single CpG analysis as it increases the extent of common findings between studies, and is more powerful as it reduces the multiple testing problem inherent in epigenetic whole-genome association studies (EWAS). In addition, results driven by single erroneous probes are removed. We have used multiple publicly available Infinium 450K data sets to generate a consensus list of DMRs for age, supporting the hypothesis that aging is associated with specific epigenetic modifications. The consensus aging DMRs are significantly enriched for muscle biogenesis pathways. We find a massive increase in VMRs with age and in regions of the genome associated with open chromatin and neurotransmission. Old age VMRs are significantly enriched for neurotransmission pathways. EWAS studies should investigate the role of this interindividual variation in DNA methylation, in the age-associated diseases of sarcopenia and dementia.

## Introduction

Epigenetic marks hold promise as biomarkers for stratifying patients for intervention in diseases with environmental and developmental causality (Gluckman *et al*., 2009). Unlike DNA biomarkers, epigenetic markers are affected by both the patient’s inherited genotype (Gibbs *et al*., 2010) and environmental exposures (Heijmans *et al*., 2008; McGowan *et al*., 2011). The epigenetic change that has attracted the most attention from translational scientists is DNA methylation (Bock, 2009; Feinberg, 2010). DNA methylation marks have been shown to be disease relevant (Martino & Prescott, 2011; Volkmar *et al*., 2012) and present before clinical symptoms of disease (Godfrey *et al*., 2011). Consequently, efforts to discover DNA methylation marks associated with a wide range of diseases have been initiated including epigenome-wide association studies (EWAS) (Ng *et al*., 2012). These studies are aided by advances in technologies to assay genomewide DNA methylation patterns such as the Illumina Infinium HumanMethylation450 BeadChip Array™ (Infinium 450K).

Infinium 450K arrays are able to measure methylation at more than 450 000 single CpGs (Bibikova *et al*., 2011). They are a cost-effective solution for surveying multiple samples and hence have achieved widespread usage. Infinium 450K profiles of 4242 samples were deposited in public repositories by 15 April 2013. Two of the substantial bioinformatics challenges in identifying biologically important differentially methylated regions (DMRs) are the massive multiple testing problem inherent in analysing more variables (> 450 000 CpGs) than observations (typically 10–100s samples) (Bock, 2009) and the possibility of aberrant values at a minority of probes due to cross-reactivity and polymorphisms in CpG sites (Chen *et al*., 2013). DNA methylation at individual CpGs has been shown to be correlated over short chromosomal distances using high density measures of the methylome (Eckhardt *et al*., 2006). We suggest that grouping contiguous CpGs into comethylated regions will add power and reduce false-positive results driven by one problematic probe. As the Infinium 450K measures CpGs in a relatively sparse and irregular fashion, it is unknown how much cis-effect can be discerned.

In this study, we show a strong negative association between correlation of methylation values between CpGs and the distance between them. We develop a region discovery method tailored to Infinium 450K methylation data, for differentially methylated region (DMR) and variably methylated region (VMR) discovery.

Methods combining neighbouring CpGs have been developed for other platforms. Jaffe *et al*. (2012a,b) published methodologies for detection of regions with contiguous differentially or variably methylated CpGs on the CHARM platform; they term their approach ‘bump hunting’. Jaffe *et al*.’s approach is theoretically applicable to Infinium 450K arrays, but would only be useful for 20% of the array. The reason for this is the sparse and spatially irregular nature of the CpGs typed on the array compared with a higher density approach such as CHARM. Infinium 450K arrays are widely used in EWAS studies, and thus, the region detection dealing with their sparse and irregular nature has utility. To our knowledge, the only method specifically designed for Infinium 450K analysis is called MethyAnalysis and deposited in bioconductor (http://www.bioconductor.org/packages/2.11/bioc/html/methyAnalysis.html). We were, however, unable to find a manuscript describing it. The tool uses a sliding window approach and is unable to provide a significance value for each region, making it difficult to rank or to compare the regions. Region detection is complementary to approaches such as Horvath *et al*. (2012) and Hannum *et al*. (2013) who determine CpGs with similar levels of methylation across and within samples regardless of their genomic position. Their comethylation approach is suitable for Infinium 27K data, while we found that region-centric analysis was not practical on the Infinium 27K array (data not shown). It has been reported that DNA methylation patterns change as a function of age (Bocklandt *et al*., 2011; Alisch *et al*., 2012; Bell *et al*., 2012; Heyn *et al*., 2012b; Hannum *et al*., 2013) and that the methylomes of identical twins ‘drift apart’ as they age (Fraga *et al*., 2005; Boks *et al*., 2009). We conducted a meta-analysis using our more specific and powerful region discovery methodology and a collection of seven previously published Infinium 450K data sets to discover a consensus list of regions whose methylation changes with age and the extent of interindividual variability in advancing age. We show a remarkable overlap between age-related DMRs from the same stage of the life course in multiple data sets, attesting the specificity of our methodology. We also show that VMRs become more common and more extreme with advancing age. As the DMRs and VMRs show significant enrichment for DNase hypersensitivity regions and biological pathways (such as neurotransmitters), we suggest that they reflect targeted environmental exposures rather than a stochastic methylome-wide drift.

## Results

### Correlation of methylation values between neighbouring probes in Infinium 450K data

Studies using genomewide methylation profiling techniques such as bisulphite sequencing and CHARM have found strong correlation of methylation levels within CpGs with genomic regions of less than 1–2 kb between them. Both these methods offer relatively complete and uniform coverage of the genome. Infinium 450K by contrast covers only a fraction of the genome in an irregular fashion. Distances between neighbouring probes on the Infinium 450K array exhibit a long-tailed spatial distribution with values ranging from 2 bp to 2 × 10^7^ bp (Fig. S1).

We asked whether the correlation between methylation levels of neighbouring probes and their pairwise distance could be discerned from the relatively sparse and irregular Infinium 450K data. To this end, we interrogated a publicly available Infinium 450K data set that assayed peripheral blood from 78 healthy boys aged between 1 and 16 years (Alisch *et al*., 2012) (hereafter referred to as the children data set). We compared the methylation values of all possible pairs of CpGs located within every 100-kb stretch across the genome for an individual (Fig. S2). We observed a strong correlation of methylation levels for neighbouring probes (< 250 bp) (Fig. S2 inset), indicating that proximal probes on the array can be grouped into ‘regions’, and associations with the outcome of interest (in this case the age of the subject) can be studied at the region level. The correlation for probes within 1 kb is moderate at ~0.45.

### A new region-centric methodology to detect DMRs and VMRs

To detect candidate DMRs and VMRs, we first assign each CpG probe a differential statistic measuring the strength of association with age (regression beta value or *t*-statistic) or median absolute deviation (MAD) score of the CpG methylation levels for each probe on the array across all individuals of the same age group. A candidate DMR is defined as at least two spatially contiguous probes within 1-kb distance of each other and with differential statistic consistently less than the 5th (for negative associations) percentile or more than the 95th (for positive associations) (detailed description in methods). A candidate VMR is defined as at least two spatially contiguous probes within 1 kb of each other and with MAD values more than the 95th percentile. We expand candidate regions to contain more than two probes, as long as the distance between any two neighbouring probes within the region is not larger than 1 kb.

For candidate DMRs, the test statistic is the area of each region defined by the genomic distance and the differential statistic. Phenotypes are repeatedly permuted to create a null distribution of DMR areas. The candidate DMR areas are then compared with the null distribution to generate a significance value that is corrected for multiple testing using FDR.

To evaluate the significance of candidate VMRs, the VMR area (defined by genomic distance and MAD) is compared by parametric bootstrapping to a simulated data set with the same MAD and genomic distance distribution as the actual data set (detailed description in methods).

### Region discovery in data sets with common age ranges finds common DMRs

**Figure 1:**
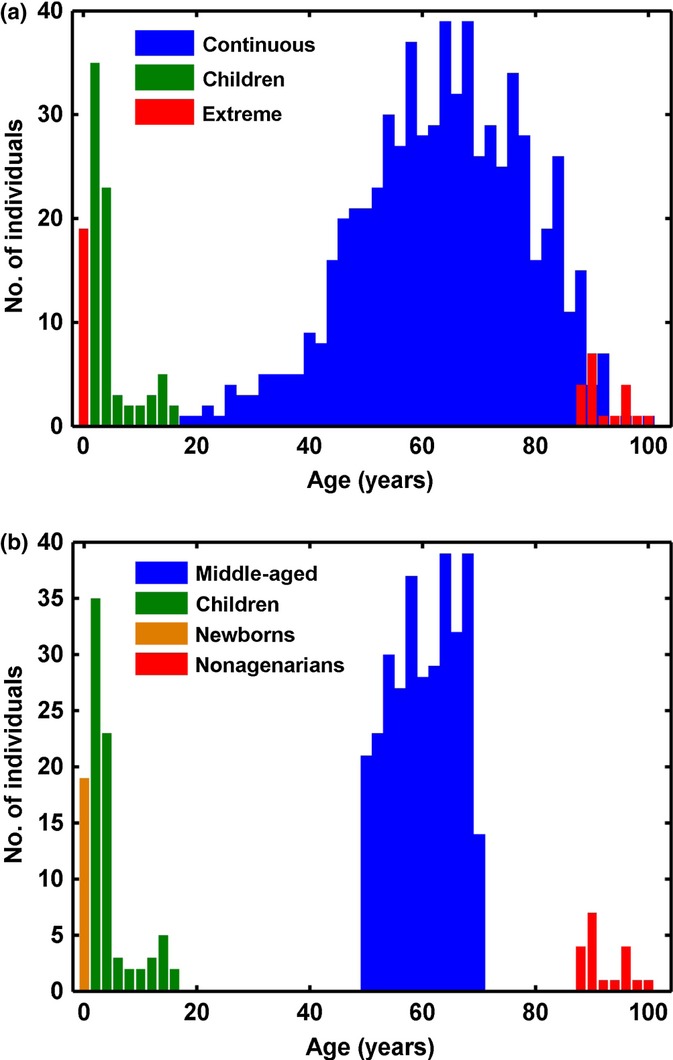
Age distribution of data used in the DMR analysis (a) and the VMR analysis (b). The children data set is in green *n* = 78 (Alisch *et al*., 2012), and the extreme (Heyn *et al*., 2012b) data set is in red in (a) *n* = 38. In (b), the data set is divided into newborns in yellow *n* = 19 and nonagenarians in red *n* = 19. The continuous (Hannum *et al*., 2013) data set is in blue *n* = 656 in (a). It is restricted to individuals between 50 and 70 years old in (b) (*n* = 319) and named ‘middle-aged’.

We identified three publicly available age-related Infinium 450K data sets. The first assayed the previously mentioned peripheral blood from 78 healthy boys between 1 and 16 years of age (Alisch *et al*., 2012) (referred to as the children data set). The second is a two-group newborn and nonagenarian peripheral bloods data set, with 19 samples in each group (Heyn *et al*., 2012b) (hereafter referred to as the extreme age data set). The third is a set of 656 peripheral blood samples from individuals aged between 19 and 101 years (Hannum *et al*., 2013), but with the vast majority of individuals in the ages between 40 and 80 years (hereafter referred to as the continuous age data set). Figure 1a displays the age distribution for each data set.

**Figure 2:**
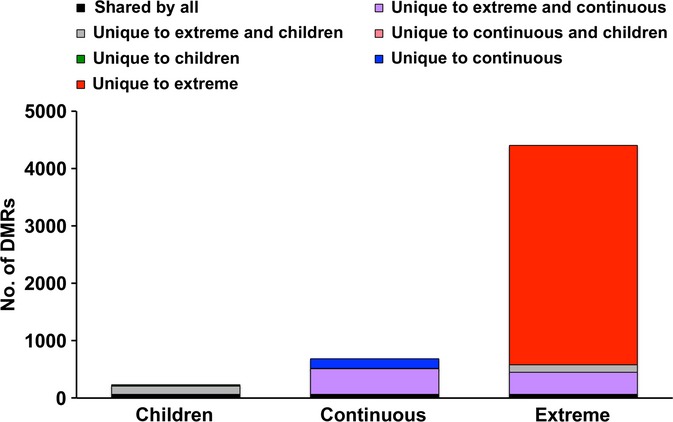
The number of age-associated DMRs returned from the three data sets. In total 234, 685 and 4405 significant DMRs were identified from the children, continuous and extreme data sets, respectively. 67 DMRs were shared by all three data sets (black), 444 DMRS were common to the extreme and continuous data set (purple), 140 DMRs were common by the extreme and children data set (grey), 5 DMRs were common to the continuous and children data sets (not visible), 22 DMRs were unique to the children data set (not visible), 170 DMRs were unique to the continuous data set (blue), and 3754 DMRs were unique to the extreme data set (red).

First, we showed that the pattern of correlation of methylation values between neighbouring probes was similar in all data sets (Fig. S3). Then, we identified 234, 685 and 4405 significant DMRs in the children, continuous age and extreme data sets, respectively [FDR < 5%, filtering regions containing probes found to cross-hybridize by (Chen *et al*., 2013)] (Fig. 2). The majority (64.8%) were hypomethylated with advancing age.

Interestingly, the number of significant DMRs identified was related to the magnitude of the age difference in the data set and not to the sample size (Fig. S4). For instance, we found a much larger number of DMRs in the extreme age data set. We suggest that this reflects the larger age range studied and the corresponding larger effect size. Interestingly, DMRs in both the children and continuous age data sets are also mostly found within the extreme data set. These overlaps are extremely significant with *P* < 10^−5^. However, the overlap between the continuous and children data sets is much more modest. The pattern of DMR overlap is reflected in the pattern of age range overlap. The extreme age data set range encompasses both the continuous and children ranges, while there is no overlap between the ranges of the children and continuous data sets (Fig. 1a). Our results suggest that although some aging DMRs are general across the life course, the majority are specific to the age range examined; that is, different regions vary in methylation during childhood than in middle age.

### Region discovery is more specific than single CpG level analysis

Data for four other aging data sets (Table 1) using the authors’ analysis results were compared with the DMRs derived above. Three of the other data sets were generated on the Infinium 27K array (Bocklandt *et al*., 2011; Bell *et al*., 2012; Horvath *et al*., 2012) and studied midlife age ranges of 20–80, 18–65 and 18–70 years, respectively, and analyses were all performed at the single CpG level. We also included the authors’ result on the continuous age data set produced using elastic net data reduction methodology, which is also likely to produce highly specific results, but via a very different mechanism to our region-centric approach (Hannum *et al*., 2013). As the four new data sets overlapped with the age range of the continuous and extreme data sets, but not of the children data set, we compared the 444 DMRs mapping to 403 genes common to the former two and not found in the latter. 89 genes in our DMR results list were also found in at least one of the new study list (Tables 2 and 3). Given that the Infinium 27K array represents only 17% of the probes on the Infinium 450K array, the 22.1% overlap observed here is remarkable.

**Table 1:** Four other aging data sets and the age ranges studied and methylation assays used

	Data	Technology	Age group
1	Bell *et al*.	Infinium 27K	32–80, 20–61
2	Horvath *et al*.	Infinium 27K	18–65
3	Bocklandt *et al*.	Infinium 27K	18–70
4	Hannum model 1 & 2	Infinium 450K	19–101

**Table 2:** Number of significant results unique to continuous and extreme returned by either a single-probe analysis or a region analysis found in at least one of the four gene lists in Table 1

Significant consensus genes from continuous and extreme data, excluding children data	No. of overlap	No. not overlapping	% Overlap
Single-probe analysis	912	8405	9.79
Region analysis	89	314	22.1

**Table 3:** Number of significant results unique to continuous and extreme returned by either a single-probe analysis or a region analysis found in at least two of the four gene lists in Table 1

Significant consensus genes from continuous and extreme data, excluding children data	No. of overlap	No. not overlapping	% Overlap
Single-probe analysis	232	9085	2.49
Region analysis	39	364	9.70

To assess the specificity of region discovery compared with a single-probe analysis, we performed a single-probe analysis on the aging data sets mentioned earlier and compared the gene list from this analysis with the results from the four additional age-related data sets. We found that only 9.8% of the significant genes obtained from a single-probe analysis were found in the four previous studies (Table 2), which is lower than the 22.1% overlap achieved by performing region discovery on the original three data sets. This suggests that an increase in specificity is achieved with region discovery (22.1% overlap to the other data sets with compared with 9.8% overlaps to the other data sets Table 2). This trend is more pronounced when the gene lists from region discovery or single CpGs analysis are compared with genes found in at least two of the new data sets (Table 3). The consensus is 9.7% for region discovery and only 2.5% for the single CpG analysis.

One possibility for the concordance of the age-related DMRs from each data set could be that region discovery is biased towards detecting particular regions on the arrays independent of phenotype. To check for such a bias, we applied the method to a study comparing DNA methylation differences in the whole blood of fifteen sets of twins (30 individuals) discordant for breast cancer (Heyn *et al*., 2012a). As the twins are perfectly age matched, no age-specific differences would be expected. We found 1338 DMRs that were different between the paired cases and controls in the breast cancer data set, and we compared this DMR list with each of the age-related DMR lists identified earlier (i.e. children, continuous and extreme age data sets). Only 4.9–6% of the age-related DMRs were also found in the cancer study.

### DMRs are found in genes implicated in aging by other mechanisms

GenAge is a high-quality, human-curated database of genes whose function is implicated in the aging process (Tacutu *et al*., 2013). In March 2013, the GenAge database contained 285 human genes corresponding to 6363 probes on the Infinium 450K array. The DMR lists from each of the three data sets studied were significantly enriched for the GenAge set on the array (Table 4), suggesting that differential methylation may reflect or cause differential transcription during aging.

**Table 4:** Overlap of genes in individual DMR lists with the GenAge gene list

Data set	No. of genes/DMRs	No. of corresponding probes on array	No. of GenAge overlap genes	No. of overlap probes on array	*P*-value
GenAge data set	285	6363	–	–	–
Children DMR list	234	913	6	26	0.005
Continuous DMR list	685	3842	14	97	9.55 × 10^−5^
Extreme DMR list	4405	28690	64	531	0.028

### Interindividual variation in methylation becomes more common and more extreme with advancing age

**Figure 3:**
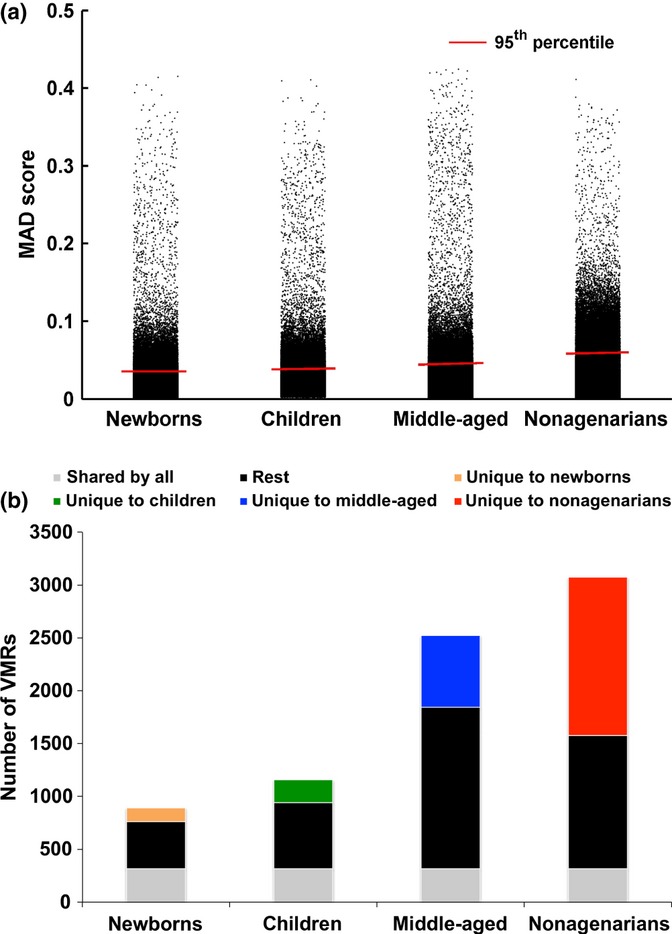
(a) Interindividual variability increases with age across the 4 age group populations. Box plot and distribution of CpG MAD scores across newborns, children, middle-aged people and nonagenarians. (b) The number of VMRs returned from the 4 data sets. 892, 1157, 2523, 3075 significant VMRs were returned from the newborn, children, middle-aged and nonagenarian data sets, respectively. 314 VMRs were shared by all four data sets (bottom grey), 129 were unique to newborns (orange), 215 were unique to children (green), 681 were unique to middle-aged (blue), and 1498 were unique to nonagenarians (red).

We discovered VMRs in the children data set, the middle-aged group that includes only 319 individuals aged between 50 and 70 years from the continuous age data set, and the newborns and nonagenarians separately from the extreme age data set (Fig. 1b). We observed that the 95th percentile MAD score for the nonagenarians was 1.5× higher than for the newborns and children and 1.3× higher than for the middle-aged (Fig. 3a). The nonagenarian group contained the most number of significant (< 5% FDR) VMRs, despite a smaller sample size than the children and middle-aged data sets (Fig. 3b). Again, the number of significant VMRs identified was independent of sample size, but dependent on the age of the subjects (Fig. S5). Also variation between the genders was predicted to account for at most 11% of the significant VMRs identified (data not shown).

### Methylation quantitative trait loci (meQTLs) may explain many of the VMRs common across all age groups, but less of those unique to each

We hypothesized that the 314 VMRs common to all the age groups may be influenced by polymorphisms common in the population as the pervasive effect of the genotype on methylation patterns has been definitively demonstrated (Gibbs *et al*., 2010; Zhang *et al*., 2010; Bell *et al*., 2011). We queried the VMRs and flanking regions for known SNPs with maximum MAF > 5%. A probe is deemed SNP free if there are no SNPs within the probe (50 bp) as well as in the 100-bp flanking region from both ends of the probe. We defined a region as SNP free if all the probes contained within it are SNP free. At the region level, 17% of VMRs common across all age groups were SNP free, compared with 21% of unique newborn VMRs, 34% of unique children VMRs, 27% of unique middle-aged VMRs and 30% of unique nonagenarian VMRs (Table 5).

**Table 5:** Comparison of percentage of SNP-free regions between VMRs shared by all and individual unique age group VMRs

	VMRs shared by all	Unique newborn VMRs	Unique children VMRs	Unique middle-aged VMRs	Unique nonagenarian VMRs
No. of SNP-free regions	54	27	74	186	454
Total no. of regions	314	129	215	681	1498
% SNP-free regions	17.2	20.9	34.4	27.3	30.3

Therefore, we suggest that a proportion of VMRs occurring in only one age group are likely to be in response to individual environment rather than a genotype-driven effect. The age-group-stable VMRs were associated with 192 genes, and these were enriched in various antigen processing and presentation pathways involving the major histocompatibility complex (MHC) (Table S2). This is consistent with their putatively more genotype-driven influence, as the genes involved in these processes are typically in more polymorphic regions of the genome.

### Biological pathways are significantly enriched in aging DMRs and age-specific VMRs

**Figure 4:**
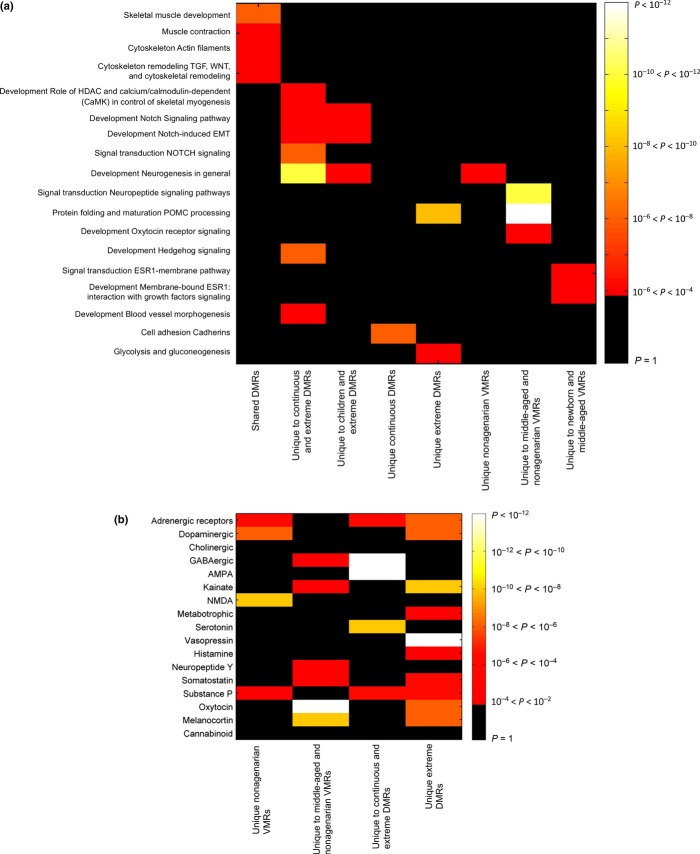
(a) Heat map of pathway enrichment results. Gene lists are indicated on the *x*-axis and pathways on the *y*-axis. Cells are coloured for *P*-value of enrichment. (b) Heat map of neurotransmitter category enrichment results. Neurotransmitter categories are indicated on the *y*-axis and region lists on the *x*-axis. Cells are coloured for *P*-value of enrichment.

**Figure 5:**
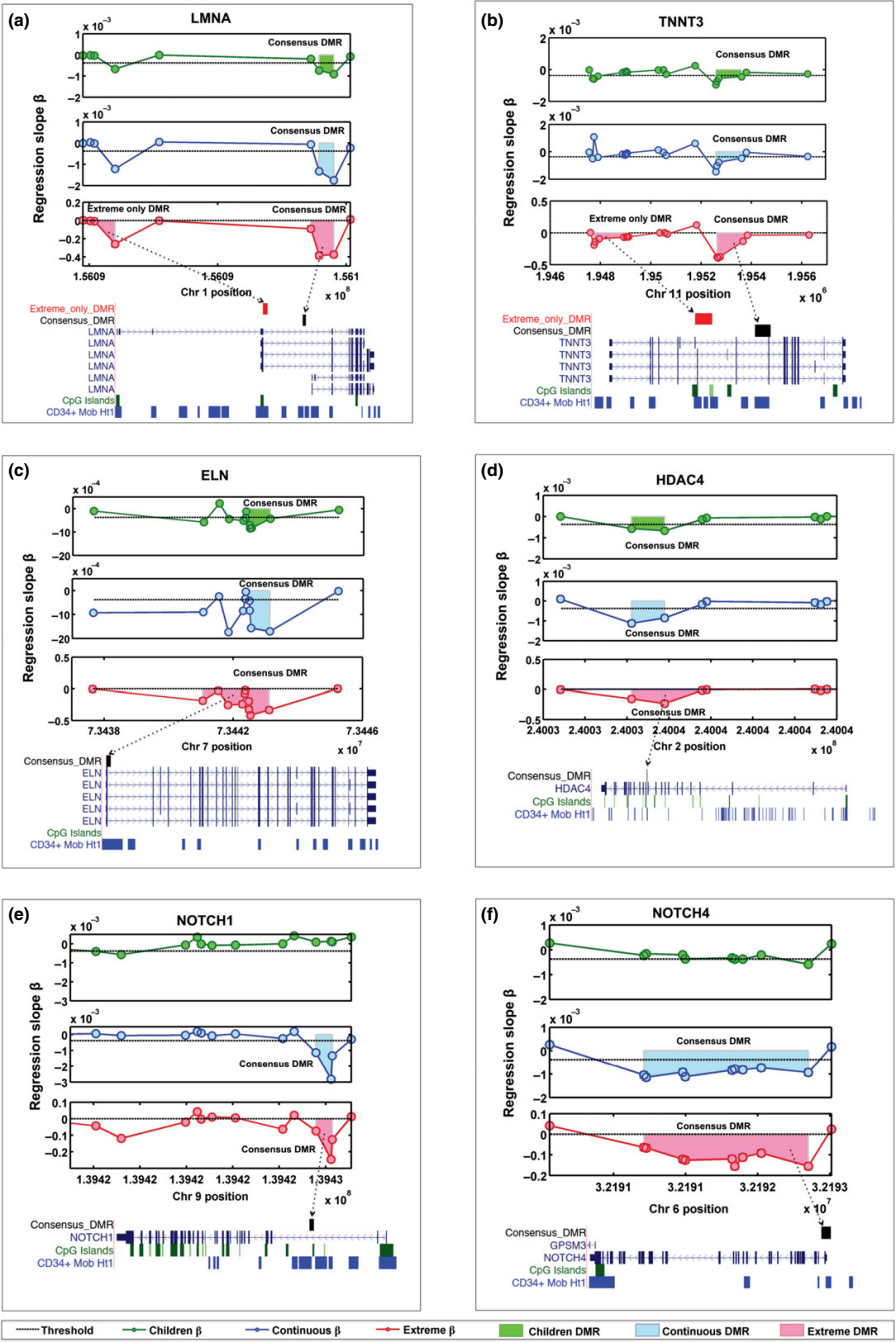
Schematic diagrams of example age-associated DMRs. The *x*-axis denotes chromosomal position, the *y*-axis denotes regression beta against age. The circles represent individual CpG probes on the Infinium 450K array. The shaded regions represent the area of significant DMRs. Also shown are the regions with respect to gene regions, CpG islands and DNase 1 hypersensitivity hot spots of CD34+ mobilized cell line. The three data sets are represented by three panels, the children data set is the top panel and represented in green, the continuous data set is the middle panel and represented in blue, and the extreme data set is the bottom panel and represented in red. DMRs within genes involved in muscle development: LMNA (a), TNNT3 (b), ELN (c), HDAC4 (d); and in Notch signalling NOTCH1 (e) and NOTCH4 (f) are shown.

The gene networks significantly enriched for DMRs (controlling for array content, see methods) that occur in all aging data sets are concerned with skeletal muscle (Fig. 4a). DMR-containing genes in these processes are *ACTA2*, *TNNT3*, *ELN*, *HDAC4*, *LMNA*, *CALR*, *MYLK* and *CD44* (Fig. 5 for examples). Mutations in the nuclear structural protein *LMNA* cause the premature aging syndrome Hutchinson–Gilford progeria, and aberrant splicing of *LMNA* is implicated in normal aging (Scaffidi & Misteli, 2006). All three age groups returned a significant DMR in the first intron of the shorter splice variant of LMNA. The extreme data set also had a significant DMR in the first exon of the shorter variant (Fig. 5a).

An enriched pathway in both the continuous and extreme DMRs is the Notch signalling pathway. Both the *NOTCH1* and *NOTCH4* receptors contain DMRs in the continuous and extreme data sets (Fig. 5e,f).

**Figure 6:**
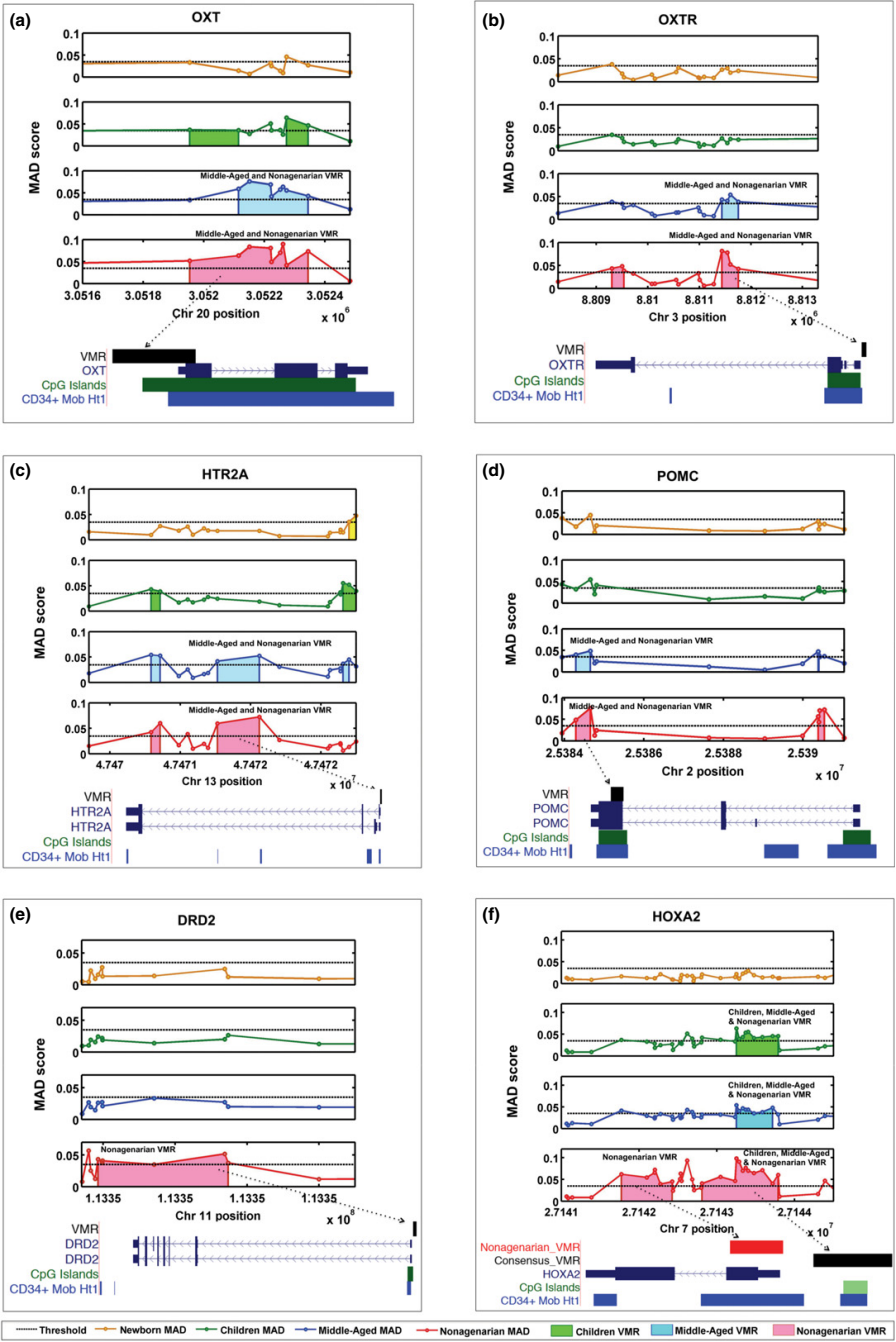
Schematic diagrams of example VMRs. The *x*-axis denotes chromosomal position, the *y*-axis denotes MAD. The circles represent individual CpG probes on the Infinium 450K array. The shaded regions represent the area of putative VMRs. Significant VMRs are illustrated together with gene regions, CpG islands and DNase 1 hypersensitivity hot spots of CD34+ mobilized cells. The four data sets are represented by four panels, the newborn data set is the top panel and is represented in orange, the children data set is the second panel and represented in green, the middle-aged data set is the third middle panel and represented in blue, and the nonagenarian data set is the bottom panel and represented in red. VMRs within genes involved in neurotransmission OXT (a), OXTR (b), HTR2A (c), POMC (d), DRD2 (e) and HOXA2 (f) are shown.

Neuropeptide signalling pathways predominate in the DMRs and VMRs of the older age groups (Fig. 4a, Table S2). When the number of CpGs mapping to neurotransmitter genes on the Infinium 450K array is controlled for (see methods), neurotransmitter genes contain DMRs and VMRs much more frequently than expected by chance in the DMRs unique to extreme (*P* < 10^−16^), unique to continuous (*P* = 0.004) and unique to continuous and extreme (*P* < 10^−16^), and similarly in the VMRs unique to nonagenarians (*P* = 5 × 10^−10^), unique to middle-aged (*P* = 0.009) and unique to middle-aged and nonagenarian (*P* = 4 × 10^−10^). However, they are not significantly enriched in the shared DMRs or VMRs, or in the unique to children DMRs, or unique to newborns or children VMRs (Table S3). Notable among the specific neurotransmitter pathways enriched for DMRs and VMRs among the older subjects are GABAnergic, AMPA, vasopressin, oxytocin, kainite, NMDA, serotonin and melanocortin signalling (Fig. 4b). Examples of the genes driving these pathways in the VMRs can be seen in Figure 6a–e. For all six examples, the area of the VMR increases with age.

### Age-associated DMRs and age-specific VMRs occur preferentially in regions of open chromatin and CpG island borders

We used the ENCODE data (Bernstein *et al*., 2012; Dunham *et al*., 2012) to investigate whether age-associated DMRs and VMRs are also found in open chromatin regions, as detected by DNase I hypersensitivity. All DMR and VMR lists are significantly colocated in DNase 1 hypersensitivity hot spots from blood cell line data deposited by the ENCODE consortium specific for CD20 and CD34+ mobilized cell types, but not in TH1, GM06990 and GM12865 cell lines (Table 6 and Table S4).

**Table 6:** Overlap of DMRs and VMRs with DNase I hypersensitivity hot spots of CD20 and CD34+ mobilized blood cell types

	No. of CD34+ mobilized DNase I hypersensitivity hot spots	No. of CD20 DNase I hypersensitivity hot spots	Total no. of regions in list	*P*-value for CD34+ overlap	*P*-value for CD20 overlap
Extreme DMR	3827	3227	4405	< 10^−16^	< 10^−16^
Children DMR	188	153	234	< 10^−16^	1.26 × 10^−7^
Continuous DMR	615	427	685	< 10^−16^	4.41 × 10^−13^
Nonagenarian VMR	2037	1579	3075	< 10^−16^	0.00242
Middle-aged VMR	1663	1335	2523	< 10^−16^	1.65 × 10^−5^
Newborn VMR	604	509	892	< 10^−16^	3.57 × 10^−7^
Children VMR	903	743	1157	< 10^−16^	< 10^−16^
Infinium 450K array	27736	26895	55003	–	–

There was significant enrichment for the various DMR and VMR classes in CpG island location categories; however, no overriding pattern was obvious (Fig. S6). CpG islands themselves were actually enriched for continuous DMRs, and enrichment in open seas was seen in the children DMRs and VMRs.

## Discussion

### Region detection increases power and specificity in EWAS studies

We describe a methodology for detecting differentially methylated regions in data from Infinium 450K arrays that are very widely used in epigenetic studies. We show that correlation of pairwise CpG methylation with distance can be detected on the sparse and irregular Infinium 450K arrays (Fig. S2). Region detection is more specific than single CpG analysis because it returns much fewer DMRs and increases the extent of common findings between studies by more than twice that seen in CpG level analyses (Tables 2 and 3). As region detection reduces the number of variables to be tested against phenotype, the method is more powerful and reduces the multiple testing problem. Table S6 (Supporting information) uses an example of a two-group test for differential methylation by age in the extreme data set. Assuming an effect size of two, CpG level analysis would have 39% power to detect differentially methylated CpGs with a Bonferroni-corrected *P*-value < 0.05, while region detection would have 61% power. Finally, as multiple differentially methylated CpGs are required to evidence a region, we predict that the effect of erroneous probes such as those detected by (Chen *et al*., 2013) will be minimized. However, due to the spacing of Infinium 450K probes across the genome, only 76% probes on the array can be included in a region (Table 1); therefore, we suggest that region discovery is run alongside classical single-probe analysis to maximize the discovery of candidate biologically important DMCpGs or DMRs. Single CpGs with significant associations between methylation level and phenotype should be treated with more caution than that due to significant DMRs because our results show that single CpG analyses are less specific.

### A distinct set of regions are differentially methylated with age

When applied to public data sets (Fig. 1), region detection found DMRs that were consistent in the same age ranges across studies (Fig. 2). This finding that the same DMRs change in multiple studies is in concordance with the finding of Horvath *et al*. (2012) who also demonstrated that their aging module found in an adult population can also be found in children. This suggests that a distinct set of regions are influx across the life course rather than a genomewide stochastic change (supported by Bell *et al*. (2012) who replicated 38% of their age-related DMRs from middle age in an independent sample set of young adults and by Alisch *et al*. (2012) who showed 85% of their paediatric age-associated loci had changed in a similar direction with age in adulthood albeit with a smaller rate of change).

### Pathways differentially methylated with age are involved in muscle biogenesis and neuronal signalling

The DMRs that were consistent across the data sets were very significantly enriched for pathways involved in muscle development and functioning (Figs 4a and 5). Decline in human muscle mass and strength (sarcopenia) is a hallmark of the aging process; whether methylation changes in genes such as *LMNA, TNNT3, ELN* and *HDAC4* are a cause or consequence of this process merits investigation. The DMRs were also enriched for Notch signalling (Fig. 4a and 5). The Notch pathway is a highly conserved arbiter of cell fate decisions and is intimately involved in neural development, neurogenesis, neuritic growth, neural stem cell maintenance, synaptic plasticity and long-term memory in both the developing and adult brain. There is age-related decline in Notch signalling (Seib *et al*., 2013) and expression (Kondo *et al*., 2011). Notch signalling may underlie the enrichment in systems development, especially nervous system development processes for age-methylated loci shown by (Alisch *et al*., 2012).

### Interindividual variation in methylation increases with age, especially in neuronal signalling pathways

Interestingly, and in concordance with other findings (Fraga *et al*., 2005; Martin, 2005; Bjornsson *et al*., 2008; Boks *et al*., 2009), but in disagreement with some (Gordon *et al*., 2012), we find that interindividual variation increases substantially in old age (Fig. 3a–b). Older-age VMRs showed remarkable enrichment for genes involved in neurotransmission (Figs 4a, b and 6). Some of these genes have been shown to be methylated in response to environment. For instance, *POMC* methylation can be altered by alcohol intake in mice (Govorko *et al*., 2012) and humans (Zhang *et al*., 2012) and with phenotypes such as obesity (Kuehnen *et al*., 2012). Methylation in *OXTR* has also been shown to change in response to acute psychosocial stress in humans (Unternaehrer *et al*., 2012). *OXTR* methylation has also been associated with autism (Gregory *et al*., 2009), but variation within normal human aged population has not, to our knowledge, previously been shown. Differential methylation of *HTR2A* has been shown in chronic fatigue syndrome (CFS) subjects compared with controls as a result of interaction between genotype factors and stress response mediated through cortisol (Falkenberg *et al*., 2011).

It is possible that reduced function of maintenance and proofreading enzymes allow more stochastic change to occur during aging. However, the remarkable colocation of DMRs and VMRs in DNase 1 hypersensitivity regions from blood (Table 6) and in genes involved in distinct biological pathways (Fig. 4a and 4b) suggests a nonrandom process. Rakyan *et al*. (2010) also found that age-associated DMRs co-associated with bivalent chromatin domain promoters. However, we were unable to repeat results showing that CpG island shores were enriched for age-demethylated CpGs, while CpG islands were depleted (Alisch *et al*., 2012).

### Limitations of the study

The interpretation of our results is limited by the fact that we are studying different individuals at the different age groups. In addition, we do not know the genotype of the individuals involved. Many regions variably methylated across individuals may be driven by genotype. Bjornsson *et al*. (2008) saw that methylation changes in older age tended to be similar within families, suggesting an effect of genotype even in dynamic methylation patterns. However, VMRs unique to one age group are less likely to be genotype-driven. In agreement with this, VMRs shared between the age groups are more likely to be near frequent SNPs than VMRs unique to a particular age group (Table 5). A caveat is that this is an extrapolation from minor allele frequencies present in dbSNP and not in the individuals under study. Therefore, although we cannot exclude the possibility that the nonagenarians that were studied happen to be more genetically heterogeneous, we suggest that the increased interindividual variability in old age is due to the effect of environment.

Another limitation is that the tissues studied in the children were peripheral blood mononuclear cells; in the newborns, cord blood leucocytes; in the continuous age data set, whole blood; and in the nonagenarians, peripheral blood leucocytes. Blood cell counts were not available; however, variability in the relative amounts of white blood cell types within individuals and across age groups may drive some of the DMRs and VMRs (Lam *et al*., 2012). If our thesis that some of the VMRs especially at later ages are driven by environment is correct, we have to assume that these environmental effects would have their impact in the blood. As examples, oxytocin is released into the blood in response to stressful and social stimuli (Matsuzaki *et al*., 2012), and POMC methylation has been observed in the blood associated with weight loss caused by anorexia nervosa (Ehrlich *et al*., 2010).

### Extensions of the methodology

The region detection method we present here is specifically designed for Infinium 450K data. However, it could be used for other types of methylation data especially when sparsity and irregular coverage is an issue. As CpGs are inherently nonuniformly distributed in the genome, irregularity will always be a feature of methylation data. Even though the method is optimized for Infinium 450K data, we expect that it can be applied to reduced representation bisulphite sequencing (RRBS) data or other sequencing-based approaches by fine-tuning the parameters for clustering CpGs. Conventional sliding window methods for sequencing data coerce the data into fixed window sizes. This method eliminates having to impose an arbitrary constant size for each region. Further, by using the area of the region as the test statistic, it provides a more robust assessment of the significance of DMRs.

## Conclusions

We propose a new method for detecting differentially and variably methylated regions on Infinium 450K arrays. Grouping individual CpGs typed on an Infinium 450K array into comethylated regions reduces the number of variables that are tested against a clinical observation in EWAS, and thus increases available statistical power (Table S6) and decreases false positives. We have used multiple publicly available Infinium 450K data sets to generate a consensus list of regions that are differentially methylated with age, supporting the hypothesis that aging is associated with specific epigenetic modifications. We are able to show a massive increase in interindividual variability of methylation levels by age and in targeted regions of the genome, suggesting the effect of environment causes divergence in the methylome profiles over the life course. Based on the results presented, the role of DNA methylation in sarcopenia and dementia should be investigated via EWAS.

## Experimental procedures

### Study populations

We used publicly available Infinium 450K data sets from three age-related studies GSE30870, GSE36064 and GSE40279 with 38, 78 and 656 samples, respectively, and a breast cancer-related study GSE37965 that contains 30 methylation profiles from 15 sets of twins discordant for breast cancer (Heyn *et al*., 2012a). In addition, we directly used the results from four other age-related methylation studies (Table 1). All analyses were performed directly on the preprocessed data available online. In addition, we removed regions containing more than 20% cross-hybridizing probes.

### Correlation of methylation values with distance in Infinium 450K data

To determine the relationship between the correlation of methylation values with pairwise CpG distance, we first found CpG pairs across every 100-kb stretch for each chromosome. For each CpG pair, we recorded the distance between them and their methylation values (using an individual’s methylation data from the children data set). Then, we sorted the distances in ascending order, and we binned the distance with approximately equal numbers of pairs in each bin and computed the median distance of each bin. Then, we calculated the correlation of methylation values of the pairs in each bin using Pearson’s correlation. Finally, we plotted the median distances against the correlation values for all bins.

### Identifying DMRs in Infinium 450K data

To determine an optimal distance threshold (named *L*) to define methylated regions, it is necessary to balance the requirement for the region to be small enough to show at least modest correlation of methylation values against the requirement for the region to be large enough to maximize the total number of probes that can either form new regions or be included into existing regions. Within 1-kb distance between neighbouring probes, the correlation is at ~0.45 or smaller (Fig. S2 inset). Therefore, we varied *L* between 250 bp and 1 kb and compared the coverage. As we increased *L*, the number of clusters decreased, while the number of clustered probes increased (Table S5), suggesting that increasing *L* mainly expands existing regions rather than creates new clusters. With *L* at 1 kb, 76% of all probes on the array can be grouped into 55 003 regions based on distance.

We use the following regression model to formalize the relationship between genomic-position-dependent methylation, covariates and age (Equation 1):

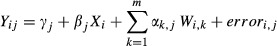
(1)

Here, *Y*_*i,j*_ refers to the methylation measurement at genomic position j for individual i. *X*_*i*_ represents age. *β*_*j*_ measures the strength of association between methylation levels at position j and age *X*_*i*_. *W*_*i,k*_ and *α*_*k,j*_ refer, respectively, to the covariates (e.g. technical batch effects or gender) and their corresponding regression coefficients. However, we did not include covariates, so W was omitted. *γ*_*j*_ is the expected mean value of *Y*_*i,j*_ when all *X*_*i*_ is 0 (controls). (The Beta statistic is not to be confused with a beta value that denotes percentage methylation as measured on Infinium arrays. To avoid confusion, we have referred to Infinium values as percentage methylation.) Let q1 and q2 be the 95th and 5th percentile for all probes with positive and negative beta values, respectively. We thus define DMRs as sets of two or more spatially contiguous probes (each pair within a maximum distance of 1 kb) all with *β*_*j*_ > q1 or all with *β*_*j*_ < q2.

### Assigning significance to DMRs

To assign statistical significance to candidate DMRs, we use as our test statistic the area of each region, defined by the genomic distance and the beta. We permute the outcomes *X*_*i*_ of the original data set and rerun the region identification procedure to identify regions and areas that would be found by chance. We repeat this for *K* simulations to create the null area distribution. Therefore, we can calculate the significance for each observed region *r*, by comparison with the null area distribution. The empirical *P*-value for each region is computed according to Equation 2:

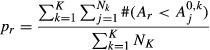
(2)where *A*_*r*_ is the observed area, 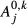
represents the *j*th null area *A*^0^ in the *k*th simulation, and *N*_*k*_ represents the total number of null areas found in the *k*th simulation. To account for multiple testing, we use the *P*-value distribution to estimate the false discovery rate (FDR) and to compute *q*-values (defined as the minimum FDR at which the test may be called significant) for each region.

### Assigning significance to VMRs

We calculate the MAD score of the CpG methylation levels for each probe on the array across all individuals of the same age group, that is, children. A candidate VMR is defined as at least two spatially contiguous probes within 1 kb of each other and with median absolute deviation values more than the 95th percentile. To evaluate the significance of the VMRs, we used a parametric bootstrapping approach. We assume that the MAD values are generated from a first-order autoregressive (AR1) process. Using Burg’s method [62], we fit this model to all regions containing at least 50 probes to obtain estimates of the coefficient *ρ* and standard deviation:

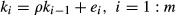
where *k*_*i*_ is the log-transformed MAD value at position *i*, and *e*_*i*_ is generated from *N*(0,Σ). We averaged the parameter estimates and used it to generate synthetic MAD distributions. For each simulated MAD distribution, we randomly selected *x* number of regions (*x* = actual observed VMR size) and computed the null areas. The empirical *P*-value is obtained by comparing the actual observed VMR areas with the null areas from all simulations according to Equation 2.

### Statistical and permutation analysis

Areas are approximated using trapezoidal numerical integration. For the analysis of multiple data sets, we applied the lowest 95th percentile value as the cut-off for both DMR and VMR detection. All permutations and simulations were performed 100× unless otherwise stated. To account for multiple testing, we used the *P*-value distribution to determine the q-values. All statistical analyses and simulations were carried out in Matlab (MathWorks) and R.

### Significance of overlaps

For a two-group enrichment test, we used the hypergeometric test (one-tailed) to determine the *P*-value for the overlap. For intersects of more than two groups, we performed simulations to determine the empirical *P*-value. For each list, we randomly selected *x* number of null regions from the background set (*x* = observed size), and we determined the overlap size expected by chance from 10^5^ repetitions. The empirical *P*-value is calculated as the number of times the actual observed overlap is smaller than the null overlap divided by the total number of simulations.

### Comparison with four other age-related data set results

For the single-probe analysis, we performed linear regression and computed the probewise t-statistics and *P*-values for the regression coefficients, and we applied the same 95th percentile and FDR < 5% cut-offs used in the region analysis, in the single-probe analysis. In separate comparisons, we calculate the overlap between:

1. the gene lists obtained from the DMR analyses with the results from the four other aging data sets and
2. the gene lists obtained from the single CpG analysis with the results from the four other aging data sets;

by scoring one for each gene if it appeared in both the original three data set’s results and the other four studies’ results.

### GenAge overlap significance analysis

The 55 003 regions can be identified on the array form the background list from which DMRs were generated. To compute the overlap significance of DMRs with the GenAge data set, we first determined the total number of probes represented by the 285 GenAge genes in the background list, and we determined the number of GenAge genes appearing in each of our DMR list and the total number of probes represented by these genes. We computed the overlap significance for each list using a hypergeometric test (one-tailed).

### SNP analysis

For each probe on the array, we queried dbSNP database for SNPs with MAF > 5% located within the probe and within 100-bp flanking regions from the ends of the probe.

### Gene ontology and pathway analyses

Gene ontology and pathway analyses were performed in GeneGo (Metacore 5.0) (http://thomsonreuters.com/products_services/science/systems-biology/). Here, we used as our background list, the representation of all genes on the Infinium 450K array, to test for enrichment in our significant DMR/VMR lists. And results with significant association were determined from an exact hypergeometric distribution test (one-tailed) and were corrected for multiple testing using Benjamini–Hochberg FDR.

### Neurotransmitter enrichment analysis

To avoid the problem of overrepresentation of neurotransmitter pathways in the databases, we created our own collections of neurotransmitter genes limited to only genes encoding the peptides themselves and the receptors. We segregated these collections to specific ligands. A list of 146 neurotransmitter ligand and receptors was identified from literature. To compute the overlap significance of the DMR and VMR lists with the neurotransmitter list, we first determined the total number of probes represented by the neurotransmitter genes in the background list of 55 003 regions, and we determined the number of neurotransmitter genes appearing in each of the DMR/VMR lists and the total number of probes represented by these genes in the list. The *P*-value for general neurotransmitter gene enrichment for each region list is computed using a hypergeometric test (one-tailed). The list of 146 neurotransmitter genes can be grouped into 17 functional categories (Fig. 4b). We determined the enrichment of each DMR/VMR list for each neurotransmitter subcategory using the same analysis.

### CpG island enrichment analysis

For individual DMR/VMR lists, we determined the total number of probes belonging to each of the six genomic categories (Fig. S6), and we repeated it for the background list of 55 003 regions. The *P*-value for enrichment of the region lists with each genomic category is computed by a hypergeometric test (one-tailed).

### DNase I hypersensitivity hot spots enrichment analysis

We obtained the DNase I hypersensitivity hot spot data for peripheral blood CD20 and CD34+ mobilized cell types from the ENCODE project (University of Washington). We determined the overlap of each hot spot list with the background list of 55 003 regions. Similarly, we determined the overlap size for each DMR/VMR list. We used a hypergeometric test to compare the overlap found in the background and DMR/VMR list to determine the significance of DNase I hypersensitivity enrichment.

### Power calculations

For a single-probe analysis, ~450 000 tests can be performed. For a region analysis, there are a total of 55 003 regions and thus 55 003 tests. We use the Bonferroni correction to correct for multiple testing. Assuming the same effect size and sample size for the two analyses, we use G*Power3 (Faul *et al*., 2007) to compute the achieved power in a Wilcoxon–Mann–Whitney two-group test.

### Software

The MATLAB code is available from: https://github.com/SICS-holbrookLab/Infinium-450-DMR

## Supporting Information

**Fig. S1** CpGs typed on Infinium 450K array are irregularly spaced in the genome: Frequency distribution of distance (bp) between neighbouring CpGs on the Infinium 450K array. The inset shows a zoomed-in region of distances less than 5000 bp, where the majority of CpGs reside. **Fig. S2** Correlation of CpG methylation levels of probes within close distances is discernible of the Infinium 450K array: Pearson correlation of pairwise Infinium 450K measured methylation levels (*y*-axis) with distance (bp) (*x*-axis). The inset shows a zoomed-in region of distances < 2500 bp, where the correlation ranges from approximately 0.9 to 0.1. **Fig. S3** Correlation of CpG methylation levels with distance for different age groups: a) Pearson correlation of pairwise Infinium 450K measured methylation levels (*y*-axis) with distance (bp) (*x*-axis). b) The figure shows a zoomed-in region of distance < 250 bp. **Fig. S4** Relationship between number of significant DMRs identified and a) sample size or b) age difference in sample set. **Fig. S5** Relationship between number of significant VMRs identified and a) sample size or b) median subject age in sample set. **Fig. S6** Heat map of CpG island enrichment results. Genomic regions of varying proximity to CpG islands are indicated on the *x*-axis and region lists on the *y*-axis. Cells are coloured for *P*-value of enrichment.

**Table S1** Lists of significant DMRs and VMRs.

**Table S2** Gene ontology categories for DMR and VMR lists with *P* < 10^−4^ and FDR < 5%

**Table S3** Overall enrichment of DMR and VMR lists for neurotransmitter genes. **Table S4** Overlap of DMRs and VMRs with DNase I hypersensitivity hot spots of three other blood cell types. **Table S5** Coverage (no of probes on the array that can be grouped into regions by distance) as a function of the distance threshold. **Table S6** Comparison of power achieved using single probe and region analysis on the extreme data set.
