# Supplementary material for: Novel region discovery method for Infinium 450K DNA methylation data reveals changes associated with aging in muscle and neuronal pathways
Source: Aging Cell. 2013 Oct 22;13(1):142–55. doi: 10.1111/acel.12159 (PMC4326857; doi:10.1111/acel.12159)
Supplement: Supplementary file 1 — Fig. S1 CpGs typed on Infinium 450K array are irregularly spaced in the genome: Frequency distribution of distance (bp) between neighbouring CpGs on the Infinium 450K array. The inset shows a zoomed-in region of distances less than 5000 bp, where the majority of CpGs reside. Fig. S2 Correlation of CpG methylation levels of probes within close distances is discernible of the Infinium 450K array: Pearson correlation of pairwise Infinium 450K measured methylation levels (y-axis) with distance (bp) (x-axis). The inset shows a zoomed-in region of distances < 2500 bp, where the correlation ranges from approximately 0.9 to 0.1. Fig. S3 Correlation of CpG methylation levels with distance for different age groups: a) Pearson correlation of pairwise Infinium 450K measured methylation levels (y-axis) with distance (bp) (x-axis). b) The figure shows a zoomed-in region of distance < 250 bp. Fig. S4 Relationship between number of significant DMRs identified and a) sample size or b) age difference in sample set. Fig. S5 Relationship between number of significant VMRs identified and a) sample size or b) median subject age in sample set. Fig. S6 Heat map of CpG island enrichment results. Genomic regions of varying proximity to CpG islands are indicated on the x-axis and region lists on the y-axis. Cells are coloured for P-value of enrichment. [file acel0013-0142-sd1.pdf]

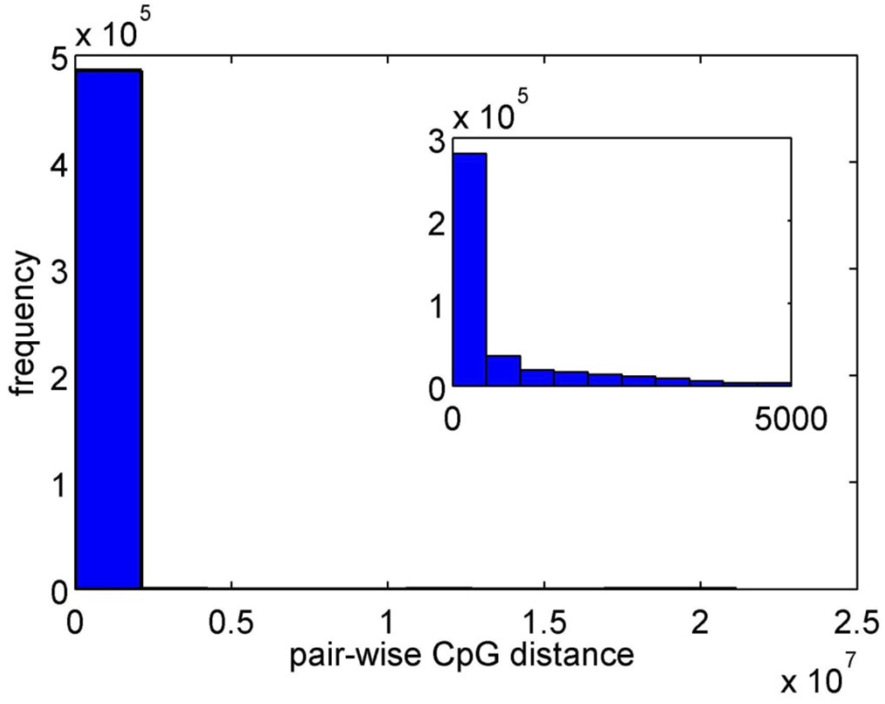

Supplementary Figure 1. CpGs typed on infinium 450K array are irregularly spaced in the genome: Frequency distribution of distance (bp) between neighbouring CpGs on the Infinium 450K array. The inset shows a zoomed in region of distances less than 5000 bp, where the majority of CpGs reside.

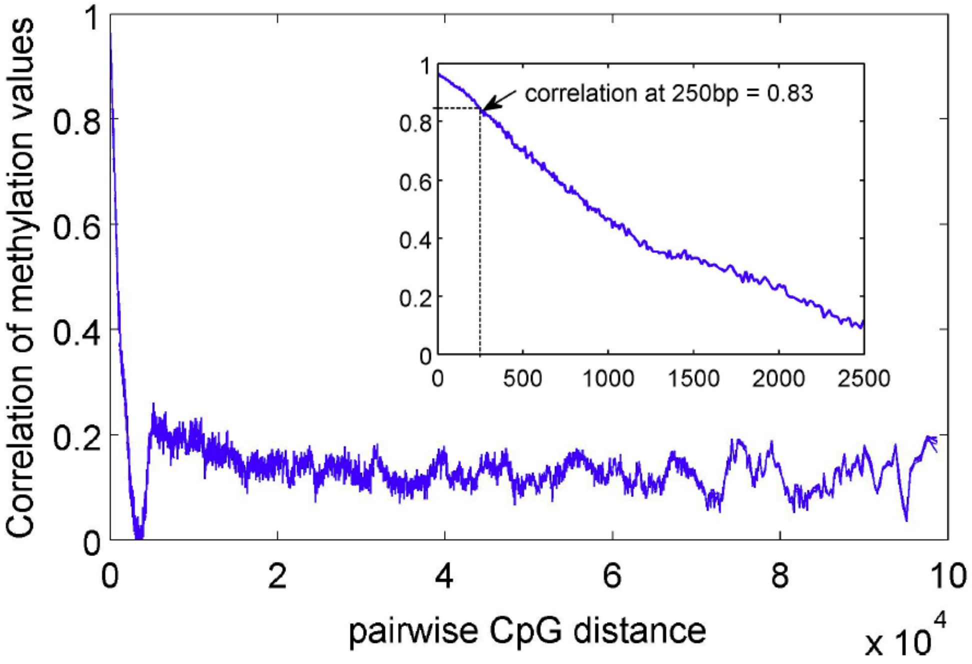

Supplementary Figure 2. Correlation of CpG methylation levels of probes within close distances is discernable of the Infinium 450K array: Pearson correlation of pair-wise Infinium 450K measured methylation levels (y-axis) with distance between the probes (bp) (x-axis). The inset shows a zoomed-in region of distances < 2500bp, where the correlation ranges from approximately 0.9 to 0.1.

a

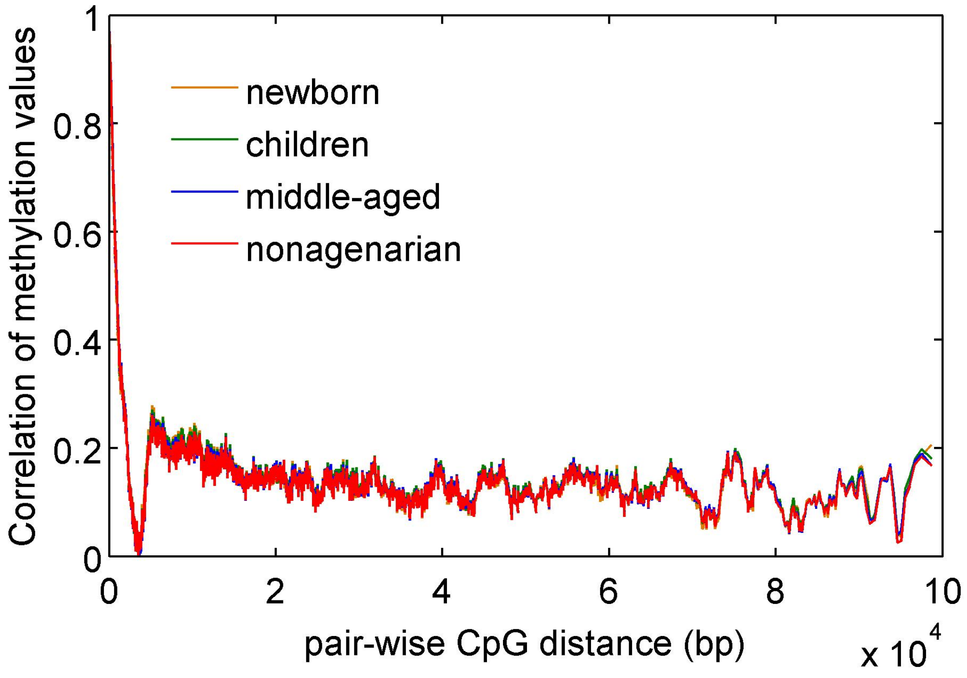

Correlation of methylation values
pair-wise CpG distance (bp)
x 10^4
newborn
children
middle-aged
nonagenarian
0
0.2
0.4
0.6
0.8
1
0
2
4
6
8
10

b

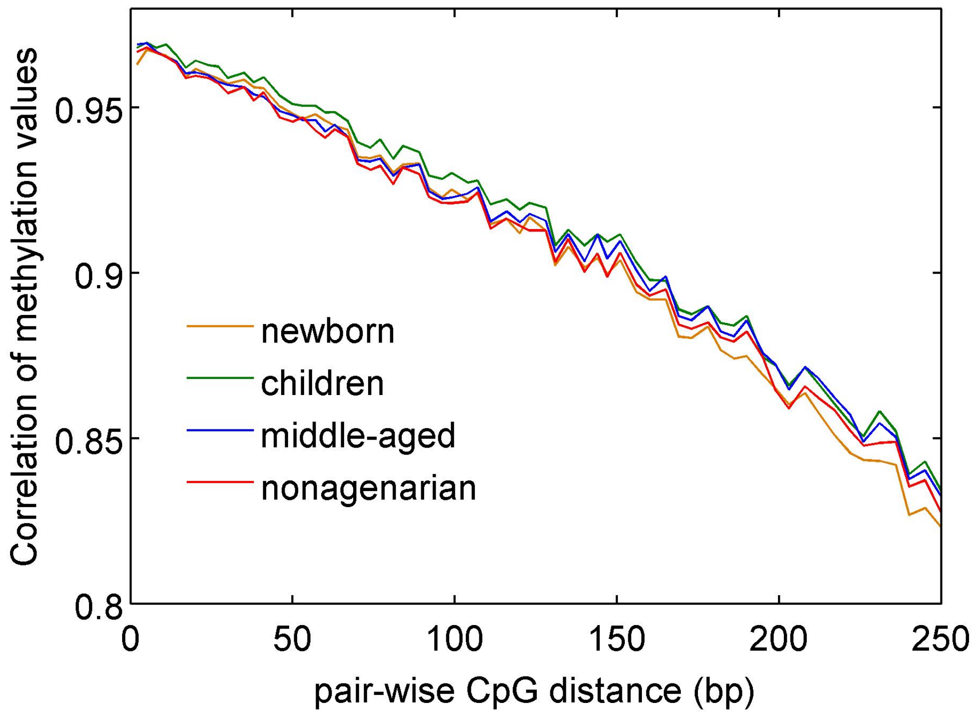

Correlation of methylation values
pair-wise CpG distance (bp)
newborn
children
middle-aged
nonagenarian
0.8
0.85
0.9
0.95
0
50
100
150
200
250

Supplementary Figure 3. Correlation of CpG methylation levels with distance for different age groups: a) Pearson correlation of pair-wise Infinium 450K measured methylation levels (y-axis) with distance (bp) (x-axis). b) The figure shows a zoomed-in region of distance < 250bp.

a

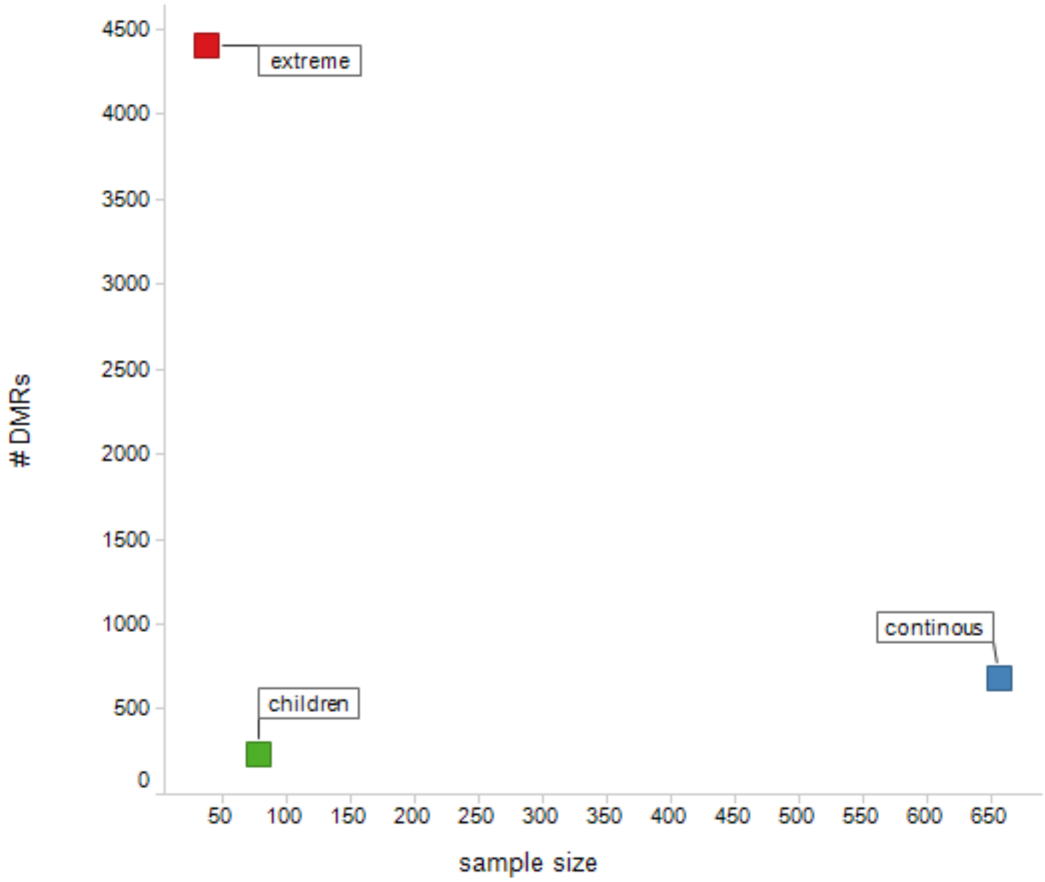

4500
4000
3500
3000
2500
2000
1500
1000
500
0
DMRs
extreme
children
continous
50
100
150
200
250
300
350
400
450
500
550
600
650
sample size

b

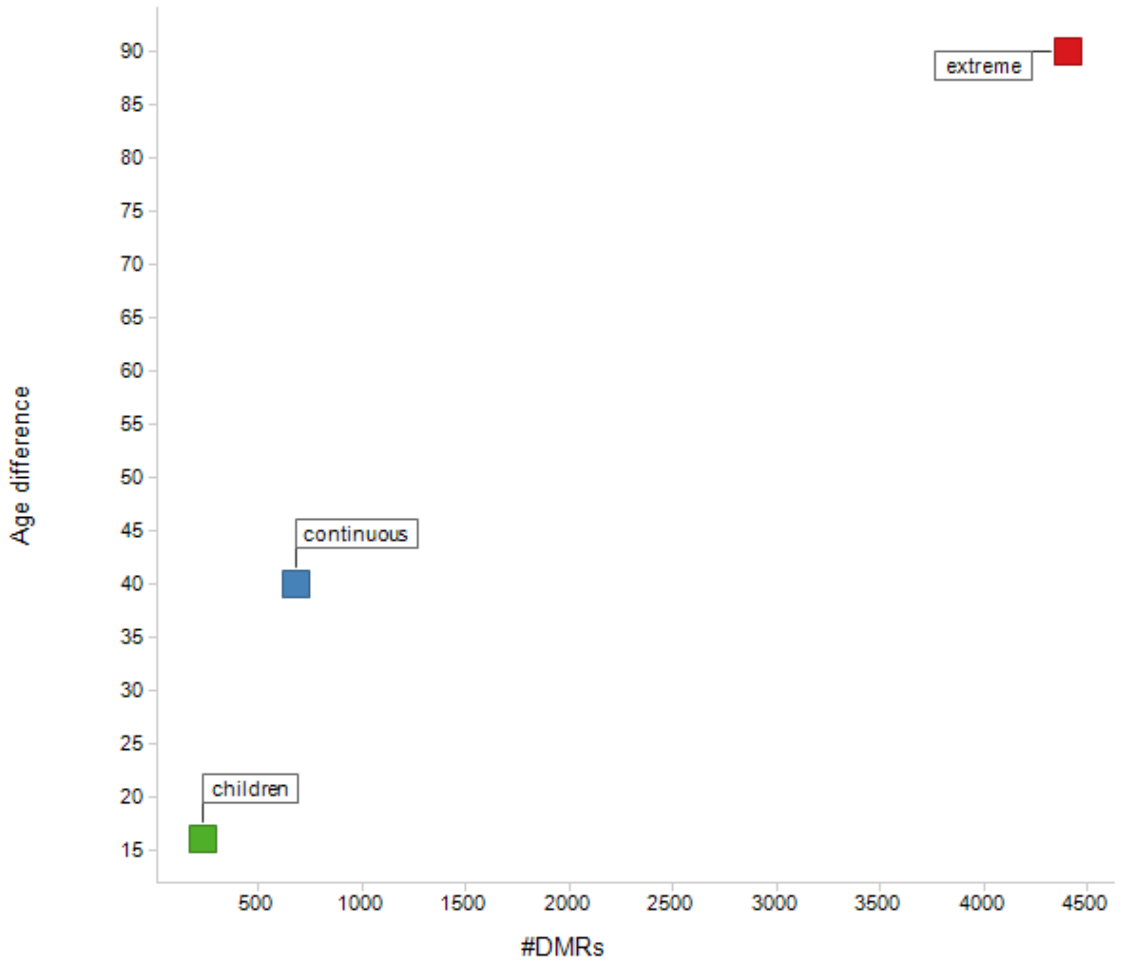

Supplementary Figure 4. Relationship between number of significant DMRs identified and a) sample size or b) age difference in sample set

a

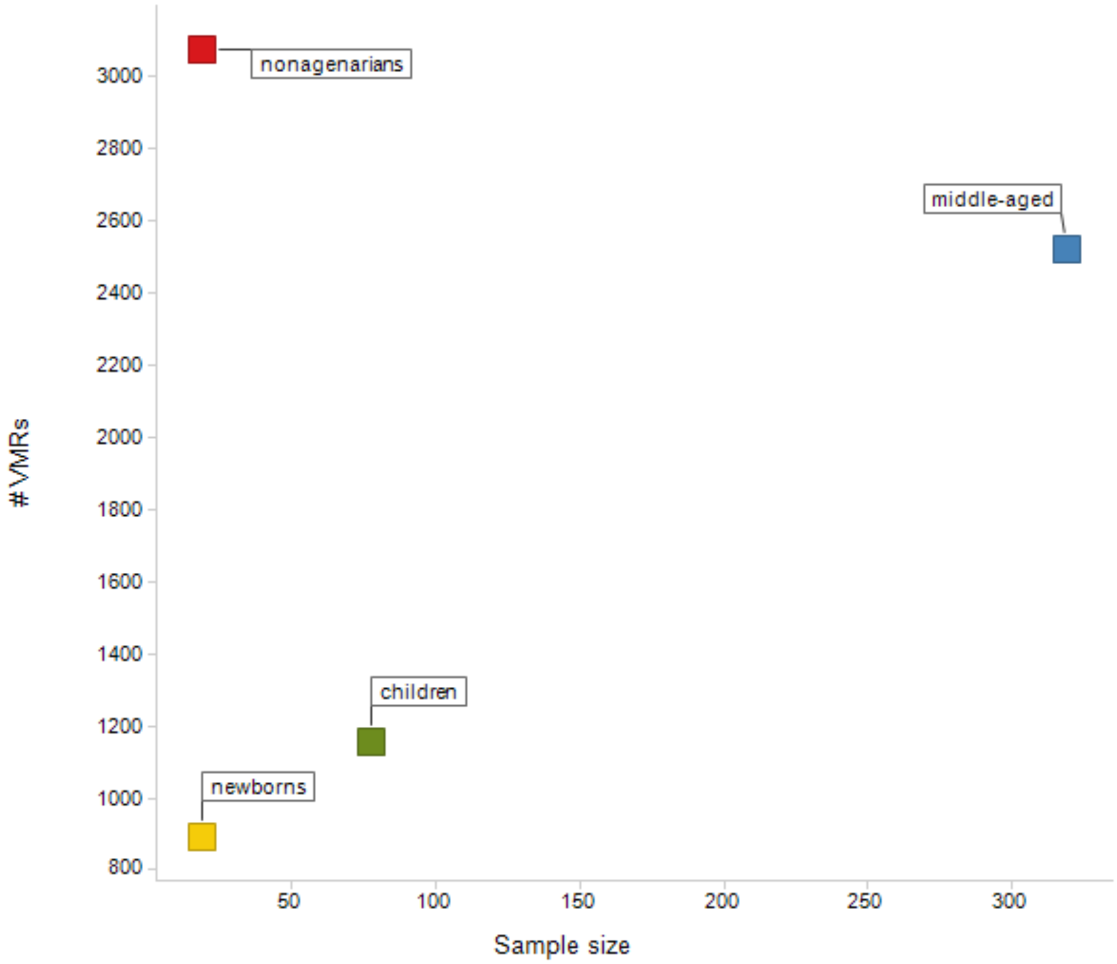
nonagenarians
middle-aged
children
newborns
VMRs
3000
2800
2600
2400
2200
2000
1800
1600
1400
1200
1000
800
50
100
150
200
250
300
Sample size

b

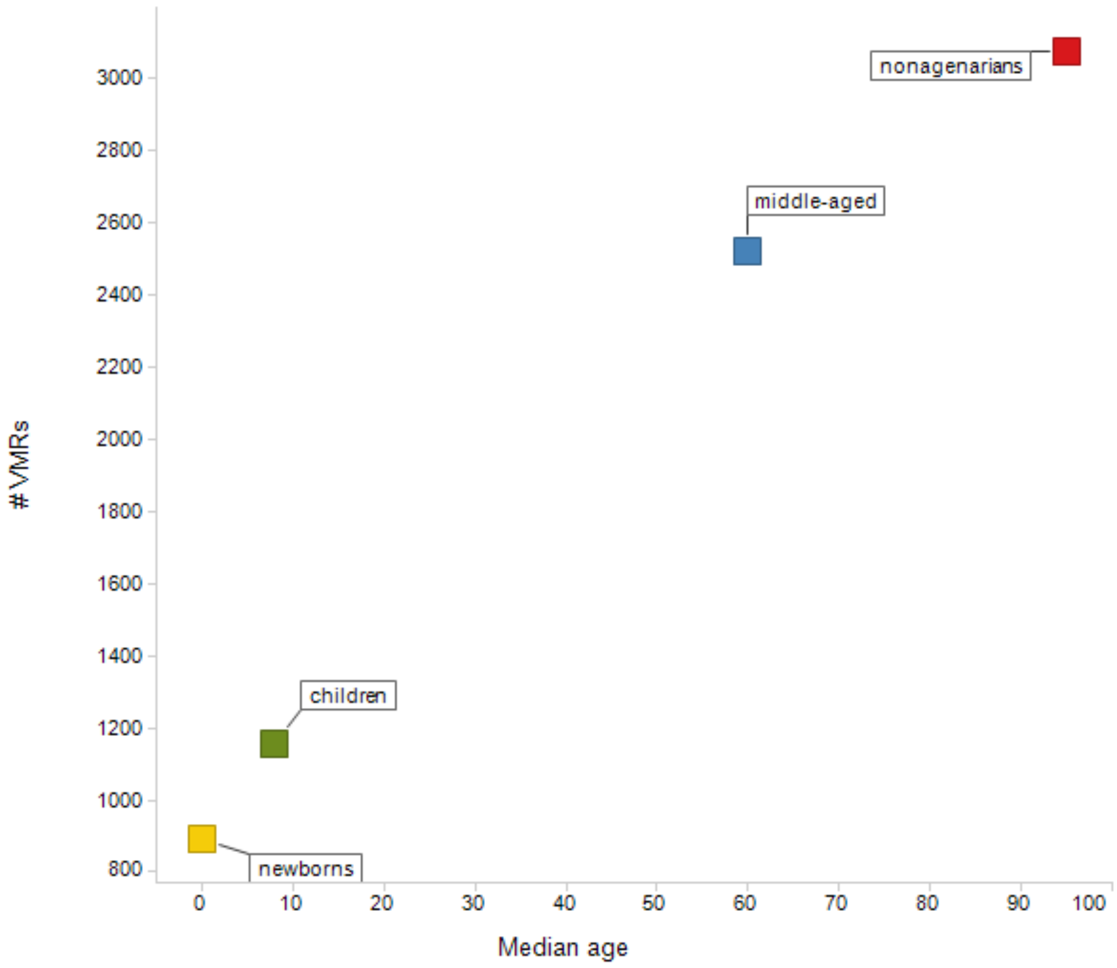

Supplementary Figure 5. Relationship between number of significant VMRs identified and a) sample size or b) median subject age in sample set

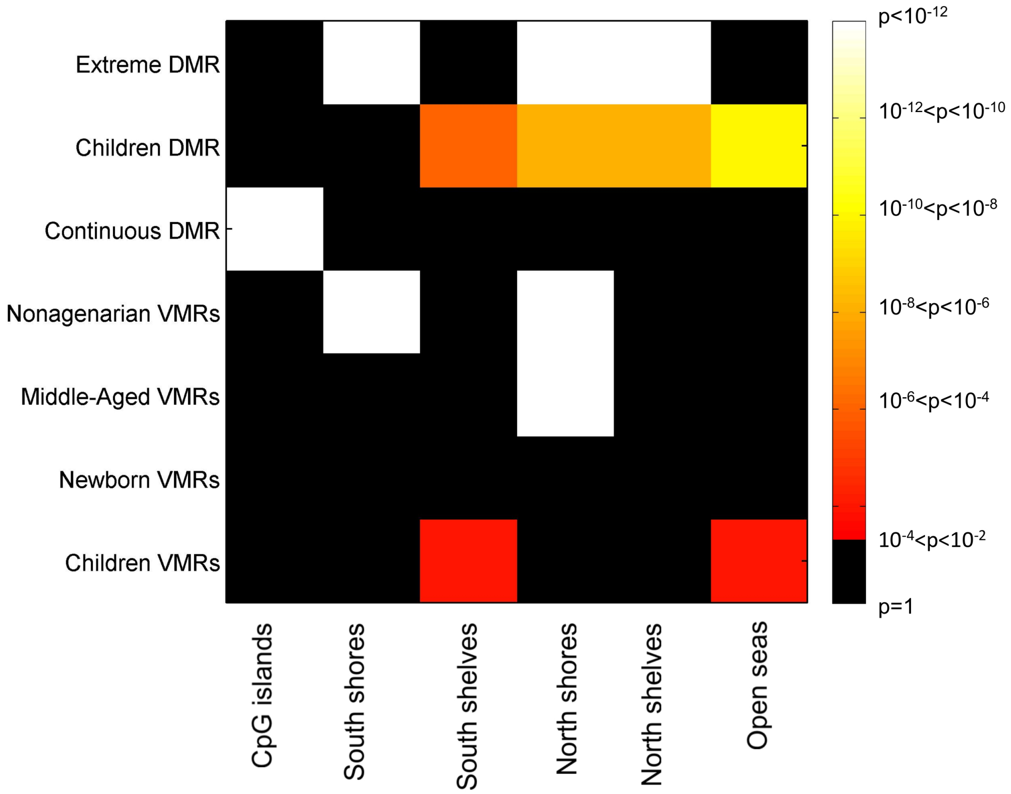

Supplementary Figure 6. Heat-map of CpG island enrichment results. Genomic regions of varying proximity to CpG islands are indicated on the x-axis and region lists on the y-axis. Cells are coloured for p-value of enrichment.
