# Supplementary material for: Novel region discovery method for Infinium 450K DNA methylation data reveals changes associated with aging in muscle and neuronal pathways
Source: Aging Cell. 2013 Oct 22;13(1):142–55. doi: 10.1111/acel.12159 (PMC4326857; doi:10.1111/acel.12159)
Supplement: Supplementary file 4 — Table S3 Overall enrichment of DMR and VMR lists for neurotransmitter genes. Table S4 Overlap of DMRs and VMRs with DNase I hypersensitivity hot spots of three other blood cell types. Table S5 Coverage (no of probes on the array that can be grouped into regions by distance) as a function of the distance threshold. Table S6 Comparison of power achieved using single probe and region analysis on the extreme data set. [file acel0013-0142-sd4.docx]

**Supplementary table 3**

|  | Overall p-value of neurotransmitter enrichment |
| --- | --- |
| VMRs unique to nonagenarians | 5.34E-10 |
| VMRs unique to middle-aged | 8.80E-03 |
| VMRs unique to children | 1 |
| VMRs unique to newborns | 1 |
| VMRs shared by all | 7.86E-01 |
| VMRs unique to middle-aged and nonagenarians | 3.99E-10 |
| VMRs unique to children and middle-aged | 1.28E-05 |
| VMRs unique to children and newborn | 1 |
| VMRs unique to children and nonagenarians | 1 |
| VMRs unique to newborn and middle-aged | 1 |
| VMRs unique to nonagenarians and newborns | 1 |
| DMRs unique to children | 1 |
| DMRs unique to extreme | 1 |
| DMRs unique to continuous | 3.62E-03 |
| DMRs shared by all | 1 |
| DMRs unique to children and extreme | 1.16E-05 |
| DMRs unique to children and continuous | 1 |
| DMRs unique to continuous and extreme | <E-16 |

**Supplementary table 4**

|  | No. of DNase I hypersensitivity hotspots | | |  | p-value for overlap | | |
| --- | --- | --- | --- | --- | --- | --- | --- |
|  | TH1 | GM06990 | GM12865 | Total no. of regions in list | TH1 | GM06990 | GM12865 |
| Extreme DMR | 2538 | 2891 | 2508 | 4405 | <E-16 | <E-16 | <E-16 |
| Children DMR | 120 | 117 | 126 | 234 | 0.0031 | 0.244 | 2.64E-05 |
| Continuous DMR | 258 | 361 | 267 | 685 | 0.996 | 0.00559 | 0.847 |
| Nonagenarian VMR | 838 | 1211 | 792 | 3075 | 1 | 1 | 1 |
| Middle-Aged VMR | 852 | 1037 | 770 | 2523 | 1 | 1 | 1 |
| Newborn VMR | 279 | 380 | 272 | 892 | 1 | 0.999 | 1 |
| Children VMR | 492 | 575 | 471 | 1157 | 0.514 | 0.110 | 0.559 |
| Infinium 450K array | 23442 | 26379 | 22532 | 55003 | - | - | - |

**Supplementary table 5**

| Maximum distance between neighboring CpGs *L* (bp) | 250 | 500 | 1000 |
| --- | --- | --- | --- |
| No. of clusters (≥ 2 probes) | 75038 | 63443 | 55003 |
| No. of probes represented by clusters | 302223 | 344055 | 371934 |
| % array | 62 | 71 | 76 |

**Supplementary table 6**

|  | No. of tests | Assumed effect size | Bonferroni adjusted α | Sample size in each group | Power |
| --- | --- | --- | --- | --- | --- |
| Single probe | 450000 | 2 | 1.11E-07 | 19 | 0.386 |
| Region | 55003 | 2 | 9.09E-07 | 19 | 0.607 |
